# TRIM Proteins and Their Roles in the Influenza Virus Life Cycle

**DOI:** 10.3390/microorganisms8091424

**Published:** 2020-09-16

**Authors:** Hye-Ra Lee, Myoung Kyu Lee, Chan Woo Kim, Meehyein Kim

**Affiliations:** 1 Department of Biotechnology and Bioinformatics, College of Science and Technology, Korea University, 2511 Sejong-ro, Sejong 30019, Korea; leehr@korea.ac.kr (H.-R.L.); kcw524@korea.ac.kr (C.W.K.); 2 Infectious Diseases Therapeutics Research Center, Korea Research Institute of Chemical Technology, 141 Gajeongro, Yuseong, Daejeon 34114, Korea; lmg1671@krict.re.kr

**Keywords:** influenza virus, ubiquitination, antiviral immunity, TRIM family

## Abstract

The ubiquitin-proteasome system (UPS) has been recognized for regulating fundamental cellular processes, followed by induction of proteasomal degradation of target proteins, and triggers multiple signaling pathways that are crucial for numerous aspects of cellular physiology. Especially tripartite motif (TRIM) proteins, well-known E3 ubiquitin ligases, emerge as having critical roles in several antiviral signaling pathways against varying viral infections. Here we highlight recent advances in the study of antiviral roles of TRIM proteins toward influenza virus infection in terms of the modulation of pathogen recognition receptor (PRR)-mediated innate immune sensing, direct obstruction of influenza viral propagation, and participation in virus-induced autophagy.

## 1. Introduction

Influenza virus is an enveloped RNA virus that harbors a genome comprising eight segments of negative-sense, single-stranded RNA encoding at least ten distinct proteins [1]. It is one of the major human respiratory pathogens that occur during seasonal or global pandemics from zoonotic infections with considerable public health and economic burdens [1,2]. Within a viral particle, genome segments are individually encapsidated with nucleoprotein (NP) to form viral ribonucleoproteins (vRNPs) complexed with the three polymerase subunits, PB2, PB1, and PA. When a virus enters the cells through recognition of the sialic acid-terminating glycan receptors by the viral hemagglutinin (HA) glycoprotein, vRNP complexes are released into the cytoplasm through the endocytic pathway, subsequently delivered into the nucleus for their replication [3,4]. At this initiation step, low endosomal pH not only stimulates membrane fusion mediated by the hemagglutinin 2 protein (HA2), but also activates the M2 proton channel, driving cytoplasmic release of vRNPs from the inner shell of the viral matrix protein 1 (M1) [5,6]. For de novo assembly of influenza viral particles, the newly synthesized vRNPs interact with M1 again in the nucleus and this complex recruits nuclear export protein (NEP, originally named non-structural protein 2 or NS2) with the ability to bind to the nuclear export receptor, chromosomal maintenance 1 (CRM1) [7,8,9,10,11]. At the final stage, viral progenies are released from the plasma membrane on which structural proteins, such as HA, neuraminidase (NA) and M2, are localized.

The ubiquitin-proteasome system (UPS) is a network of proteins dedicated to the ubiquitylation of cellular targets and the subsequent control of numerous cellular functions. Therefore, viruses must be subverted or utilized for this cellular machinery to reshape the cellular environment for establishing productive viral infection. In line with this point of view, accumulating evidence has highlighted emerging roles for the UPS during the influenza virus infection by targeting specific tripartite motif (TRIM) proteins [12,13]. The TRIM protein family is a group of E3 ubiquitin ligases which are important components of UPS and involved in various cellular functions such as innate immunity and autophagy through maintaining a critical level of crucial regulatory proteins [14,15,16,17]. Remarkably, several TRIM proteins directly target viral proteins, leading to restrictions in the viral replication either through proteasomal mediated degradation or by modulating action of target proteins [18,19]. It was elucidated that to combat this TRIM-mediated antiviral activities, several viruses have evolved to employ the strategies that hijack TRIM protein actions. This review will focus on how the influenza virus utilizes the TRIM proteins to escape TRIM-mediated antiviral action for propagation.

## 2. The Ubiquitin System

**Figure 1:**
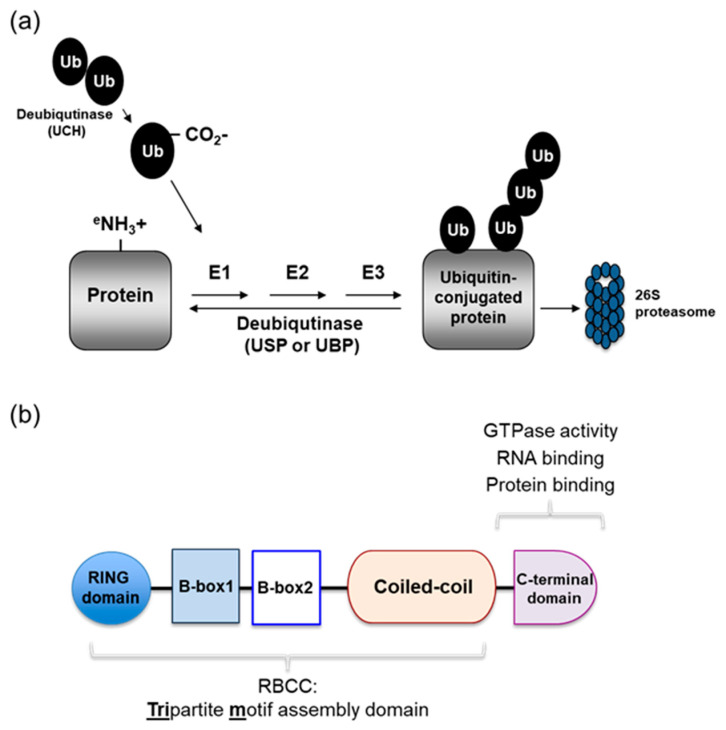
Ubiquitin modification system and schematic map of the tripartite motif (TRIM) proteins. (**a**) Cleavage of plolyubiquitin into monoubiquitin which is activated by the ubiquitin-activating enzyme, E1, followed by transfer to the ubiquitin-conjugating enzyme, E2. Finally, ubiquitin E3 ligases directly or indirectly hand over the E2-bound ubiquitin to diverse substrates. Deuibiquitinases (DUBs), such as ubiquitin specific protease (USP), catalyze the removal of ubiquitin from ubiquitin chains, leading to remodeling ubiquitin modification and antagonizing ubiquitin-driven function. (**b**) Schematic map of the TRIM family proteins. They consist of a really interesting new gene (RING) domain, B-box1 and B-box2 domains, a coiled-coil domain (CCD), and distinct *C*-terminal domain. RBCC, RING finger-B box-coiled coil.

Ubiquitination is one of the well-characterized post-translational modifications by which ubiquitin, 76 amino acid small protein, is covalently attached to lysine residues on a target protein [20]. Three cascade steps are required to induce ubiquitination that is generally destined to be connected to the 26S proteasome for degradation: First, the ubiquitin is activated by the E1 enzyme which represents adenylation of the ubiquitin *C*-terminal carboxyl group. Subsequently, this activated ubiquitin is transferred to the active cysteine site on the ubiquitin-conjugating E2 enzyme. E3 ubiquitin ligase enzymes finally transfer E2-conjugated ubiquitin to substrate proteins via forming an isopeptide bond between the *C*-terminal glycine residue of the ubiquitin molecules and ε-amino group of a lysine in the substrates [20,21] (Figure 1a). Ubiquitin can attach once (monoubiquitination) or repetitively in chains (polyubiquitination) on target proteins, consequently governing their fate, function, and regulation of additional downstream substrates [20,22]. Notably, K48-linked polyubiquitination chains are responsible for substrate degradation by the 26S proteasome, whereas K63-linked polyubiquitination plays a crucial role in the signaling pathway via modulating protein–protein interaction. Currently, seven distinct types of polyubiquitination chains have been identified based on different internal lysine residues of ubiquitin (K6, K11, K27, K29, K33, K48, and K63) and also show different fates of modified substrates. For instance, K6- and K11-linked polyubiquitination chains are involved in DNA damage and trafficking, respectively [23,24]. Ubiquitination is a reversible process by deubiquitinating enzymes (DUBs, also referred to as deubiquitinase) which have the ability to remove ubiquitin moieties from ubiquitinated substrates, leading to overall equilibrium of UPS [25]. Interestingly, these non-substrate linked poly-ubiquitin chains, called unanchored ubiquitin chains, play an important roles in several cellular responses, including innate immune response and autophagy [24,26].

## 3. E3 Ubiquitin Ligases: TRIM Family

Among the UPS components, diver E3 ubiquitin ligases that determine substrate specificity have been discovered in humans, being estimated to have more than 600 different members [20]. Given that the machinery by which ubiquitin is transferred from E2 to a substrate by E3 ligases, their superfamily is divided into three major groups: Really Interesting New Gene (RING), Homologous to E6-associated protein C Terminus (HECT) domains, and RING-in-between-RING (RBR) [27,28,29,30]. RING E3 ligases are most prevalent and act as mediators for ubiquitination of target proteins, whereas HECT and RBR E3 ligases contain a catalytic cysteine site that forms a direct thioester-bond with ubiquitin before transferring it to a substrate [20,24].

TRIM proteins have been identified as a family of RING-domain containing E3 ubiquitin ligases that are involved in various biological processes including growth, apoptosis, morphogenesis, and transcription, as well as oncogenesis. In addition, more interestingly, recent studies have highlighted their emerging roles in antiviral functions via targeting directly viral proteins, innate immune systems, or autophagy pathway [31,32,33,34]. Until now, they have been comprised of more than 80 distinct members in humans that contain a conserved three domains: an *N*-terminal RING domain, one or two B-boxes, and a central coiled-coiled domain (CCD) (Figure 1b). The RING domain appears in most of the TRIM family proteins and possesses E3 ubiquitin ligase catalytic activity, leading to modulation of the function of broad and various substrates. The B-box includes a zinc-binding motif similar to the RING domain, but its function is less well characterized. Nonetheless, current studies suggest that the B-box domain coordinates self-oligomerization, which consequently gives rise to different features with other TRIM family proteins that do not participate in the oligomerization [31]. The CCD is responsible for homo- or hetero-dimerization, as well as protein–protein interaction [31,34]. Moreover, the *C*-terminal region of the TRIM proteins generally contains one or two compositions variable in length that are classified into 11 subgroups such as Sp1A kinase and Ryanodine receptors (SPRY) domain, PRY-SPRY domain, and *C*-terminal subgroup one signature (COS) domain. Particularly, this *C*-terminal region affects subcellular localization and interaction with target proteins. For example, the SPRY and PRY–SPRY domains contribute to protein–protein interaction, exhibiting diverse roles in innate immune responses as observed in TRIM25 [31]. The COS domain generally is also associated with the microtubule of cytoskeleton network [16,31].

The TRIM proteins play a crucial role in regulating viral infection and the antiviral strategies which are classified by three broad categories: (1) modulation of pathogen recognition receptor (PRR)-mediated innate immune sensing, (2) direct obstruction of viral propagation, and (3) virus-induced autophagy. Herein, we describe these three functions of TRIM proteins in terms of intrinsic antiviral activities.

### 3.1. Antiviral Roles of TRIM-Mediated Innate Immune Responses by Influenza Virus

As noted earlier, the TRIM proteins attract attention as a positive and negative regulation of the PRR-mediated innate immune signaling pathway. There are several types of PRRs that recognize different pathogen-associated molecular patterns (PAMPs) and activate specific signaling molecules participating in innate immunity. Among them, the RIG-I-like receptor (RLR) belongs to the DExD/H-box RNA helicases and functions as an essential sensor of viral double-stranded RNA (dsRNA) or single-stranded RNA (ssRNA) in the cytoplasm of the infected cells [35,36]. In particular, among RLR members, retinoic acid-inducible genes-I (RIG-I) senses 5′-triphosphate (5′-ppp)-containing short dsRNA, while melanoma differentiation-associated protein 5 (MDA5) detects long dsRNA or aggregated viral RNA; thus, these two RLRs turn on the innate immune response associated with infection of RNA viruses as well as DNA viruses. During the normal condition, RIG-I is in an inactive state in which helicase domain interacts with its own caspase activation and recruitment domains (CARDs). Upon recognition of the 5′-pppRNA, it undergoes conformational change to expose the CARDs through K63-linked polyubiquitination by E3 ligase, followed by binding to the mitochondrial antiviral signaling protein (MAVS). In the next step, MAVS simultaneously recruits the IκB kinase ε (IKKε) and TANK-binding kinase 1 (TBK1) to activate interferon regulatory factor 3 (IRF3)/IRF7. In order to stimulate NF-κB, MAVS also engages the IKKα/β/γ complex, resulting in degradation of the NF-κB inhibitor, IκBα. This event induces translocation of NF-κB into the nucleus and promotes transcription of diverse downstream immune-regulatory genes, including pro-inflammatory cytokines and chemokines [19]. Importantly, NF-κB activation upregulates production of RLR signaling-mediated type-I interferons (IFNs) [36].

**Figure 2:**
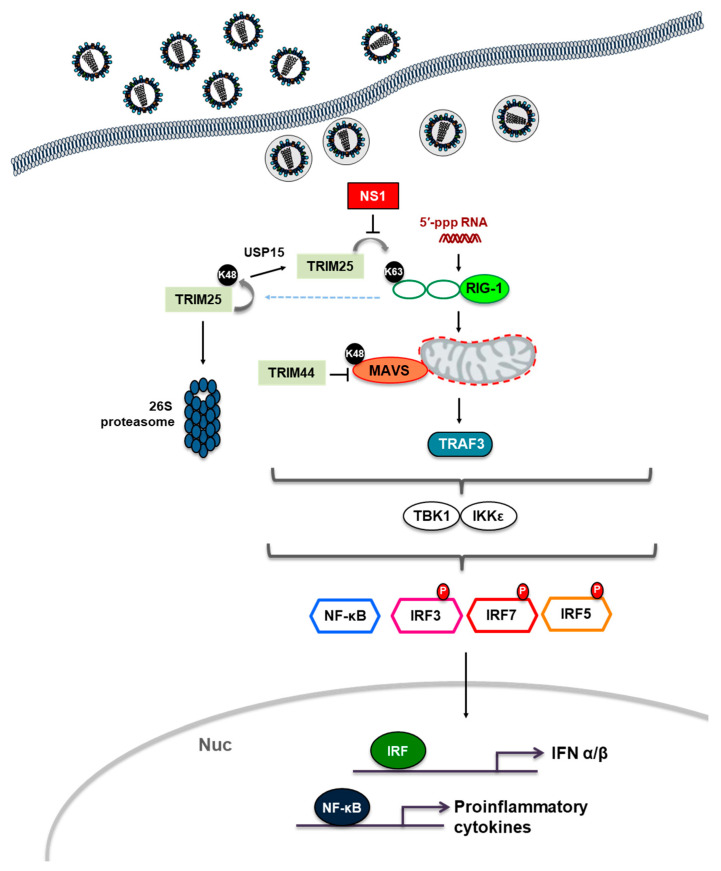
Roles of TRIM proteins in pattern recognition receptors (PRRs)-mediated immune signaling pathway. Sensing influenza viral component, herein 5′-triphosphated viral RNA or short dsRNA, by host PRRs triggers signaling pathways that promote production of type I interferon (IFN) and pro-inflammatory cytokines. Influenza virus non-structural protein 1 (NS1) combats the host retinoic acid-inducible gene I (RIG-I)-mediated immune response by targeting TRIM25. RIG-I utilizes the mitochondrial antiviral signaling protein (MAVS) and STING as adaptor molecules to activate downstream molecules, respectively, which ultimately induce the production of type I IFN and/or pro-inflammatory cytokines. TRIM44 stabilizes MAVS by inhibiting K48-linked polyubiquitination of MAVS and by preventing its degradation. Faint green square boxes represented different TRIMs and red square box indicated influenza virus NS1 protein. Nuc means nucleus.

Several TRIM proteins operate as positive regulators of PRRs-mediated innate immune signaling via targeting RIG-I, MDA5, and MAVS [19]. Among them, TRIM25 has been best characterized; it stabilizes RIG-I oligomerization and modulates antiviral activity through induction of K63-linked polyubiquitination at the K172 residue in the CARD of RIG-I [37]. Furthermore, TRIM25 synthesizes the unanchored poly-ubiquitin chains for enhancing the RIG-I function [38]. Depending on the activated RIG-I, K48-linked polyubiquitination of TRIM25 stimulates its own proteasomal degradation, thereby negatively regulating RIG-I-associated downstream signaling, whereas the ubiquitin specific protease 15 (USP15) promotes deubiquitination of TRIM25, resulting in stabilized TRIM25 to maintain the balanced innate immune response overall [39]. The crucial role of TRIM25 in antiviral signaling is retained due to the fact that nonstructural protein 1 (NS1) proteins of both influenza A and influenza B viruses antagonize RIG-I mediated antiviral signaling [40,41,42,43] (Figure 2). The NS1-TRIM25 interaction blocks K63-linked ubiquitination of CARD of RIG-I, leading to suppression of the RIG-I-mediated type I IFN signaling [40]. Notably, this antagonistic action is highly conserved among influenza A virus strains of different host backgrounds [44]. Very recently, Marcos–Villar et al. showed that influenza virus infection upregulates expression of Dot1L, which serves as a histone H3K79 methyltransferase, in an NS1-dependent manner, resulting in a decrease of the IFN-β production via suppressed TRIM25 expression [45]. Hence, these studies have strongly supported that TRIM25 functions as a bona fide antiviral factor, while NS1 is an essential virulence factor of which its function supports an efficient viral life cycle. Similar to TRIM25, which acts as a positive regulator of RIG-I-mediated signaling for enhancing type I interferon, TRIM44 inhibits the K48-polyubiquitin-induced degradation of MAVS, leading to stimulation of the type I interferon pathway via stabilization of MAVS [46]. Although the various TRIM-family proteins are involved in RIG-I-mediated immune response, TRIM25 is the only protein via targeting from the influenza virus. Therefore, further studies are required for elucidating how influenza regulates different activities of TRIMs on RIG-I-mediated immune response.

In addition to modulation of cytosolic RNA stimulate response, TRIM family proteins also regulate cyclic GMP-AMP synthase (cGAS)-STING-mediated cytosolic DNA sensing pathway. This cGAS-STING-mediated DNA sensing pathway has been recently identified as a component of the innate immune system of which functions to detect cytosolic viral DNA [47]. Upon sensing DNA, cGAS generates secondary messenger cGAMP, followed by the binding and activation of STING which triggers IRF3 phosphorylation by TBK1. After that, activated IRF3 induce transcriptional upregulation of type I IFNs [47,48]. Currently, some TRIM proteins are known to induce cGAS-STING-mediated cytosolic DNA sensing pathway. First, TRIM56 E3 ligases induced mono-ubiquitination of cGAS, enhancing its dimerization, DNA-binding activity, and cGAMP production, consequently playing a role as a positive regulator of cGAS-mediated DNA sensing pathway [49]. TRIM32 catalyzes K-63 linked polyubiquitination of STING that facilitates antiviral response [50]. TRIM14 has been shown to recruit the deubiquitinase USP14 to stabilize cGAS, enhancing the activation of type I IFN signaling against herpes simplex virus type 1 (HSV-1) [51]. Lastly, TRIM38 occurred sumoylation of cGAS and STING, leading to stabilization of them via inhibition of ubiquitin mediated degradation [52]. Given that TRIM proteins are critical effectors of the cGAS-STING-induced immune response for viral infection, it is not surprising that several viruses have evolved antagonizing strategies against these TRIM proteins’ action to ensure their replication. Interestingly, unlike other viruses, there is no report as to how the influenza A virus (IAV) subverts activities of the TRIM family that has been shown to have an effect on the cGAS-STING-induced immune response yet. Thus, further efforts are required to elucidate IAV actions which target TRIM on cGAS-STING-induced immune response.

### 3.2. Antiviral Roles of TRIM by Directly Targeting Influenza Viral Proteins

**Figure 3:**
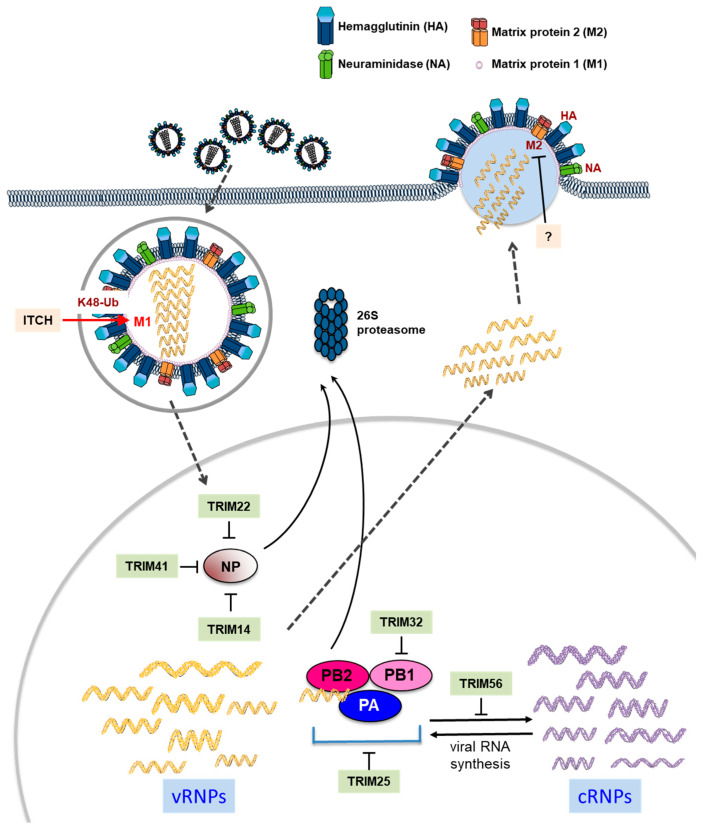
Direct antiviral effects of diverse TRIM proteins on replication of influenza virus. After receptor-mediated endocytosis of influenza virus, the viral ribonucleoprotein (vRNP) complexes are released into the cytoplasm, then delivered to the nucleus for initiation of viral RNA replication and transcription. TRIM14, TRIM22 and TRIM41 induce proteasomal degradation of the viral nucleoprotein (NP). In addition, TRIM25 and TRIM32 occurred proteasomal degradation of viral polymerase components via interaction with vRNA complex and PB1, respectively. TRIM56 inhibits viral RNA synthesis. HECT-type E3 ubiquitin ligase, ITCH, induces proteasomal degradation of matrix protein (M1), facilitating to release of vRNP into the cytoplasm.

Some TRIM proteins, including TRIM5 and TRIM22, have the ability to effectively restrict the pathogens by direct interaction with viral proteins [31]. Interferon-inducible TRIM22 has been shown to have antiviral effects against various viruses. For instance, it suppresses human immunodeficiency virus 1 (HIV-1) long terminal repeat (LTR)-driven transcription through interfering with SP-1 by binding to the HIV-1 promoter [53]. This E3 ubiquitin ligase also inhibits hepatitis B virus (HBV) by being a transcriptional repressor and encephalomyocarditis virus (EMCV) by promoting ubiquitination of 3C protease, and suppressing viral protein expression [54]. Intriguingly, a study revealed that TRIM41 binds to influenza virus nucleoprotein (NP) through its SPRY domain and then causes ubiquitination of the NP, suggesting that TRIM41 acts as an intrinsic host restriction factor to the influenza virus [55] (Figure 3). Like TRIM 41, TRIM14 also binds and degrades NP in which it effectively inhibits the formation of vRNP complex and thus RNA-dependent RNA replication [56]. Furthermore, TRIM22 interacts with NP and induces its proteasomal degradation, resulting in restriction of replication [57]. Interestingly, Isable et al. show that 2009 the pandemic H1N1 strain, as well as other H1N1 pandemic strains isolated between 1933 and 1934, were resistant to the antiviral effect of TRIM22 [58]. It is implied that the influenza virus evolutionarily developed adaptive mutation to generate favorable environment escaping TRIM22-mediated antiviral machinery. Additionally, TRIM32 ubiquitinates influenza viral polymerase components, PB1, via direct interaction, triggering its proteasomal degradation and subsequently diminishing viral polymerase activity [59]. TRIM25 has been reported to inhibit the onset of influenza viral RNA chain elongation via binding to vRNPs in the nuclei of infected cells [60]. Another TRIM protein, TRIM56, efficiently inhibits intracellular influenza virus RNA synthesis, but in an E3 ligase activity-independent manner [61]. Taken together, it is summarized that diverse TRIM proteins play a crucial role as intrinsic antiviral factors by directly impeding activities of influenza viral proteins, such as PB2, PB1, PA, and NP, composing vRNP complex with the negative-stranded viral RNA.

### 3.3. Antiviral Roles of TRIM Proteins for Influenza Virus-Induced Autophagy

Autophagy (self-eating) is an extremely conserved intracellular catabolic pathway that induces the degradation of cytoplasmic components within the lysosomes [62]. This processes is mediated by the unique double layer membrane called autophagosome and subsequently fused with lysosomes for degradation. Based on the mode of cargo delivery to the lysosome, there are three distinct classified types of autophagy, including macroautophagy, microautophagy, and chaperone-mediated autophagy. Autophagy has various physiological and pathophysiological functions; for example, dispensable intracellular protein and organelle clearance, anti-aging, cell death, tumor suppression, innate and adaptive immunity, and inflammatory disorders, as well as anti-microbial activities [62,63]. Interestingly, autophagy is able to either promote or suppress viral replication depending on viruses, cell types, and host species. For instance, dengue virus, coxsackievirus, and hepatitis C virus utilize autophagy for their replications, while Zika virus, encephalomyocarditis virus, and several herpesviruses (such as HSV-1, human cytomegalovirus, and Kaposi’s sarcoma-associated herpesvirus) evade autophagy owing to facilitation of their replication. Remarkably, autophagy has crosscurrent effects on influenza virus replication; first, different strains of IAV, including H5N1, H3N2, H9N2, and H1N1, trigger the formation of autophagy, which plays an essential role in viral replication, as well as pathogenesis [59,60,61,62,63]. Highly pathogenic avian H5N1 also enhances autophagy through inhibition of mammalian target of rapamycin (mTOR), which is a known suppressor of autophagy [64]. In addition, IAV matrix 2 (M2) ion-channel protein has been shown to inhibit a fusion process of autophagosome with lysosomes, resulting in the accumulation of autophagosome in macrophages which is critical for the survival of IAV [65]. On the other hand, IAV M2 directly interacts with the crucial autophagy protein LC3 and induces the relocalization of LC3, resulting in subversion autophagy. Eventually, the virus enhances its budding and virion stability [66]. More interestingly, a recent study has shown that IAV infection in A549 cells promotes autophagosome, leading to it acting as viral protein translational machinery [67].

It is worth mentioning that TRIM proteins have been directly linked to the multiple steps of the autophagy pathway from its induction to autophagosome formation [68,69]; First, the autophagy induction is controlled by Unc-51-like autophagy activating kinase 1 (ULK1) that leads to the assembly of large complexes containing Beclin 1. The stages of autophagosome formation are governed by Atg12-Atg5-LC3 II complexes [70,71]. TRIMs 5, 6, 17, 22, and 49 interact with ULK1 and bring to these molecules a Beclin 1 complex, leading to activation of autophagy [72,73]. Other studies have also shown that TRIMs 13, 16, 20, 21, 28, 32, and 50 are associated with ULK1 and/or Beclin 1 [70,73,74,75]. Notably, the autophagy complex formation via these TRIM proteins is not required for ubiquitination activity, while E3 ubiquitin ligases activity of TRIM 32 is needed for stimulation of ULK1 activity [70]. On the other hand, TRIM17 and TRIM59 inhibit Beclin 1-mediated autophagy induction [76,77]. The capability of TRIM proteins to regulate autophagy at the autophagosome formation level is shown by comprehensive analysis using Hela cells. Knockdown of 21 different TRIMs, out of the 67 human TRIMs, via siRNA system decreased LC3 puncta formation [72]. TRIM proteins also interact with p62, well-known cargo recognition protein and core regulators of autophagy [70]. Another comprehensive analysis study using THP-1 cells under IFN gamma (IFNγ) stimulation conditions has shown that the knockdown of 24 different TRIMs reduced the LC3 puncta, indicating that several TRIM proteins are required for IFNγ-induced autophagy as an unconventional form of autophagy [73]. In line with this point of view, a recent series of studies demonstrated that TRIM proteins have emerging roles in autophagy-associated antiviral defense and virus-induced autophagy [63,70,71,78,79]. A good example molecule of the antiviral defense is TRIM5α, which recognized the capsid protein, p24, of HIV-1 and subsequently induced the autophagy-mediated degradation [80]. Unlike HIV-1, very recently, Sparrer et al. also show that TRIM23, known as an E3 ubiquitin ligase and GTPase of ADP-ribosylation factor (ARF), promotes the dimerization of TBK1 via its GTP–GDP hydrolysis activity, resulting in the phosphorylation of selective autophagy receptor p62. Ultimately, the TRIM23-TBK1-p62 complex facilitates the influenza virus-triggered autophagy [79]. Further studies will be required to clarify the roles of TRIM proteins in autophagy during the IAV life cycle.

## 4. Antiviral Roles of Other UPS Components in Influenza Virus-Infected Cells

In contrast to the action of negative regulation regarding most TRIM proteins for the life cycle of the influenza virus, it has been reported that ubiquitination stimulates polymerase activity of the influenza virus via ubiquitination of all the vRNP components without altering the protein levels, which eventually enhance replication of the influenza virus [81]. In addition, another HECT domain-containing E3 ubiquitin ligase, ITCH, has been reported as an essential factor for influenza virus entry. ITCH interacts with M1, leading to its ubiquitination and degradation, thus facilitating release of vRNPs from viral particles [82] (Figure 3). Similarly, ubiquitination of the lysine residue at position 78 located in the M2 cytoplasmic domain has been reported to be essential for infectious virus production and for the timing of virus-mediated cell death, but without identification of the ubiquitin ligase [83]. In a cell-based assay using high-throughput screening of RNAi libraries, Liao et al. identified host deubiquitinase USP11 as a novel cellular protein, which was involved in replication of the influenza virus. Moreover, USP11 deubiquitinated mono-ubiquitination at K187 residue on the influenza NP protein, leading to inhibition of influenza virus RNA replication [84]. Taken together, these data demonstrated the capability of ubiquitination as a pivotal factor to coordinate multiple stages for influenza virus infection.

## 5. Concluding Remarks

TRIM family members serve as a commander for antiviral restriction factors and immune responses. As reviewed here, we have highlighted their critical roles in combating the influenza virus replication either by directly targeting various influenza viral proteins or by regulating either PRR-mediated innate immune response or viral infection-mediated autophagy. Despite this viral antagonism of TRIM proteins, many elegant mechanisms to evade TRIM’s action by the influenza virus have yet to be elucidated. In fact, NS1, which is the only viral protein that has been shown to subvert TRIM25-mediated immune signaling, highlighted its importance in efficient establishment of the influenza virus infection. Therefore, going forward in understanding the mechanism of antagonizing TRIMs by the influenza virus, we should address significant gaps in knowledge regarding the context-dependent regulation and specificity of both TRIMs and DUBs. By adding to our growing knowledge of TRIM proteins, we gain important insights into not only the diverse aspects of virus-host interactions, but also the inner workings of ubiquitination. It is clear that TRIM-mediated ubiquitin modification is a key to coordinating physiological and pathological processes in influenza virus-infected cells. Further insights and developments into how influenza virus thwarts TRIM-mediated antiviral mechanisms will likely yield new approaches to the treatment of viral infection.
